# Acs Symposium on Metal-Carboxylate Proteins and Synthetic Models

**DOI:** 10.1155/MBD.1995.127

**Published:** 1995


					ACS SYMPOSIUM ON
METAL-CARBOXYLATE PROTEINS
AND SYNTHETIC MODELS
Sunday a.m. Tutorial:
8:45
9:00
9:45
10:30
11:15
To be held at Anaheim, CA, USA
April 2-5, 1995
Co-organizers:
Don Kurtz and Karl Hagen
TENTATIVE PROGRAM:
Introductory Remarks Karl Hagen and Don Kurtz
Carboxylate ligation in metalloproteins and the carboxylate shift Steve Lippard
Overexpression and site-directed mutations of non-heme iron proteins, or Don Kurtz
The synthetic analogue approach
M6ssbauer spectroscopy of non-berne iron proteins Vince Huynh
Saturation magnetization of metal complexes and proteins Tad Day
Sunday p.m.:
1"30 Diiron oxo/hydroxo proteins. An overview of spectroscopy and structure, or
Vibrational spectroscopy of diiron and dimanganese proteins and models
2:10 Iron-carboxylate complexes
2:50 New, biologically relevant metal-carboxylate complexes
3:30 ENDOR and ESEEM ofdiiron proteins
4:10 Magnetization ofdiiron(II) complexes and proteins
Monday p.m.:
1:30- Crystal and molecular structures of ribonucleotide reductase and
rubrerythrin
2:10 Crystal and molecular structure of methane monooxygenase
2:50- Mechanisms of O2 reactions with the diiron sites in ribonucleotide
reductase and methane monoxygenase
3:30 02 activation by iron complexes
4:10 Fatty acid desaturases
4:50 Rubrerythrin
Tuesday p.m.:
1:30 Polyiron-oxo aggregates
2:10 Metal loading offerritin and bacterioferritin
2:50 Manganese-carboxylate clusters
3:30 Manganese catalase and synthetic models
4:10 Arginase
Wednesday a.m.:
8:30 Acid phosphatases
9:10- Urease
9:50 Phospholipase C
10:30 Dimetal aminopeptidases
11:10 Chromium-carboxylates and glucose tolerance factor
Tom Loehr
Karl Hagen
Steve Lippard
Brian Hoffman
Tad Day
P[ir Nordlund
Amy Rosenzwieg
Vince Huynh
Larry Que, Jr.
Brian Fox
Don Kurtz
Annie K. Powell
Ed Stiefel
George Christou
Chuck Dismukes
David Ash
Bernt Krebs
Andy Karplus
Li-June Ming
Richard Holz
John Vincence
More information can be obtained from:
Dr. Don Kurtz
Professor of Chemistry and Biochemistry
University ofGeorgia, USA
e-mail: kurtz@bscr.uga.edu
127

				

